# Bicistronic Vector Expression of Recombinant Jararhagin-C and Its Effects on Endothelial Cells

**DOI:** 10.3390/toxins16120524

**Published:** 2024-12-03

**Authors:** Karla Fernanda Ferraz, Lhiri Hanna De Lucca Caetano, Daniele Pereira Orefice, Paula Andreia Lucas Calabria, Maisa Splendore Della-Casa, Luciana Aparecida Freitas-de-Sousa, Emidio Beraldo-Neto, Sabri Saeed Sanabani, Geraldo Santana Magalhães, Patricia Bianca Clissa

**Affiliations:** 1 Immunopathology Laboratory, Butantan Institute, São Paulo 05585-090, Brazil; karlafernandaferraz@gmail.com (K.F.F.);; 2 Biochemistry Laboratory, Butantan Institute, São Paulo 05503-900, Brazil; 3 Laboratory of Medical Investigation LIM-56, Division of Dermatology, Medical School, University of São Paulo, Sao Paulo 05508-220, Brazil

**Keywords:** snake venom disintegrins, recombinant protein, Jararhagin-C, endothelial cells

## Abstract

Jararhagin-C (JarC) is a protein from the venom of *Bothrops jararaca* consisting of disintegrin-like and cysteine-rich domains. JarC shows a modulating effect on angiogenesis and remodeling of extracellular matrix constituents, improving wound healing in a mouse experimental model. JarC is purified from crude venom, and the yield is less than 1%. The aim of this work was to obtain the recombinant form of JarC and to test its biological activity. For this purpose, the bicistronic vector pSUMOUlp1 was used. This vector allowed the expression of the recombinant toxin JarC (rJarC) in fusion with the small ubiquitin-related modifier (SUMO) as well as the SUMO protease Ulp1. After expression, this protease was able to efficiently remove SUMO from rJarC inside the bacteria. rJarC free from SUMO was purified at the expected molecular mass and recognized by polyclonal anti-jararhagin antibodies. In terms of biological activity, both the native and recombinant forms showed no toxicity to the HUVEC cell line CRL1730 and were effective in modulating cell migration activity in the experimental in vitro model. These results demonstrate the successful production of rJarC and the preservation of its biological activity, which may facilitate further investigations into the therapeutic potential of this snake venom-derived protein.

## 1. Introduction

Disintegrins, a class of soluble polypeptides derived from various snake venoms, exhibit a remarkable degree of structural similarity and functional diversity [1,2,3,4]. Most disintegrins are thought to be derived from larger precursor proteins containing a metalloproteinase domain, although some are directly translated from mRNA sequences lacking this segment [5]. Jararhagin C (JarC) is a derivative of the PIII SVMP (Snake Venom Metaloproteinase) jararhagin and contains both disintegrin-like and cysteine-rich domains [6]. This molecule has an ECD integrin-aligning tripeptide [7] and a specialized domain that interacts selectively with the α2β1 integrin, which is predominantly found in endothelial cells [8].

Integrins are non-enzymatic transmembrane receptors that play a crucial role in facilitating cell-cell and cell-matrix interactions [9,10]. Their activation triggers various biological processes such as cell migration, adhesion, proliferation, and invasion, which are central to development, immune function, leukocyte trafficking, hemostasis, and cancer metastasis [11,12]. Interfering with integrin functions by soluble integrin ligands can have significant therapeutic effects by either agonizing or antagonizing specific integrin receptors. Previous research has demonstrated the multifunctional potential of JarC, particularly as a therapeutic agent in wound healing [13,14]. This includes applications for chronic wounds where key processes such as inflammation, angiogenesis, and extracellular matrix remodeling are impaired [15,16,17].

Extensive purification processes involving hydrophobic interaction and ion exchange chromatography are required to obtain native JarC. Despite these efforts, the final yield of JarC is about 1% of the total crude venom. Cloning and expression of this toxin in a bacterial system offers a possible solution to this low yield. However, it is a major challenge to achieve the correct structural, conformational, and functional properties of a snake venom toxin in a recombinant form. This difficulty arises from the fact that the bacterial microenvironment, e.g., in *E. coli*, differs significantly from the toxin’s native environment in terms of pH, osmolarity, redox potential, cofactors, and protein-folding mechanisms. In addition, high expression often exposes hydrophobic regions of the protein, leading to interactions with similar regions that can result in protein instability and aggregation into inclusion bodies [18,19]. The use of fusion proteins has been shown to improve the solubility and stability of recombinant proteins. Examples include glutathione S-transferase (GST) [20], Nus A [21], thioredoxin (Trx) [22], maltose-binding protein (MBP) [23], and ubiquitin-related modifying protein (SUMO) [24,25]. SUMO, a protein from *Saccharomyces cerevisiae*, belongs to the family of ubiquitin-like proteins. Although it is not found in prokaryotes, it is highly conserved in eukaryotes and plays a role in various cellular processes [26]. A major advantage of SUMO is its precise removal by the SUMO protease 1 (Ulp1), which specifically recognizes and cleaves the SUMO structure. This eliminates the risk of non-specific cleavage and enables the production of target proteins with native N-termini [24]. By ensuring clean and efficient cleavage, the SUMO protease Ulp1 facilitates the expression of functional recombinant proteins in both eukaryotic and prokaryotic systems, making SUMO an invaluable tool for optimizing protein expression [27].

To obtain sufficient amounts of JarC for further pharmacological studies, in this work, we expressed it in a bacterial expression system using the bicistronic vector pSUMOUlp1 developed by our group [28], which allowed us to obtain this toxin in recombinant active form.

## 2. Results

### 2.1. Expression and Purification of rJar-C

**Figure 1:**
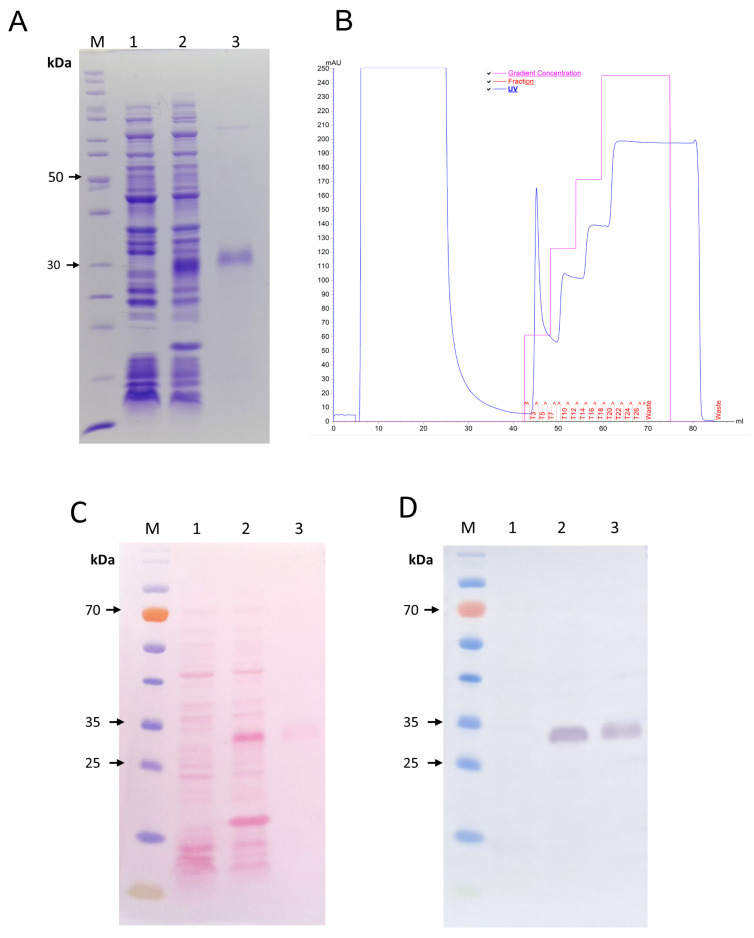
(**A**) 12% sodium dodecyl sulfate–polyacrylamide gel electrophoresis (SDS-PAGE) gel showing expression and purification of the recombinant JarC overexpressed in *E. coli* BL21 Star™ (DE3) at 37 °C. Protein was visualized on a 12.5% SDS/polyacrylamide gel under reducing conditions and stained with Coomassie blue R (Sigma-Aldrich, St. Louis, MO, USA). (**B**) Chromatogram of rJarC purification by Immobilized Metal Chelate Affinity Chromatography (IMAC). (**C**) For the analysis of rJarC expression by Western blot, proteins were separated by 12% SDS-PAGE, transferred onto nitrocellulose membrane, and stained with Ponceau red dye. (**D**) The nitrocellulose membrane was treated with an anti-His antibody for detection. For figures (**A**,**C**,**D**): The numbers on the left indicate the molecular mass markers (M). Lanes 1 and 2 show the extracts from BL21 Star™ (DE3) cells before and after induction with 1 mM isopropyl-β-D-thiogalactoside (IPTG), respectively; lane 3 shows the recombinant JarC purified by IMAC.

The pSUMOUlp1-JarC vector enabled the successful expression of recombinant JarC (rJarC) in *E. coli* BL21 Star (DE3) cells. During the expression process, the simultaneous activity of the SUMO protease Ulp1 enabled the production of SUMO-free rJarC, which was ready for purification due to the presence of a histidine tag at its C-terminus. Analysis of the purified rJarC by SDS-PAGE revealed a band corresponding to the expected molecular size of ~28 kDa (Figure 1A). This was also confirmed by Western blotting with anti-His antibodies (Figure 1D). The chromatographic profile of rJarC from IMAC purification is shown in Figure 1B, while the Ponceau-stained membrane after the Western blot transfer is shown in Figure 1C. The purified rJarC had a final yield of approximately 5 mg/L of bacterial culture.

**Figure 2:**
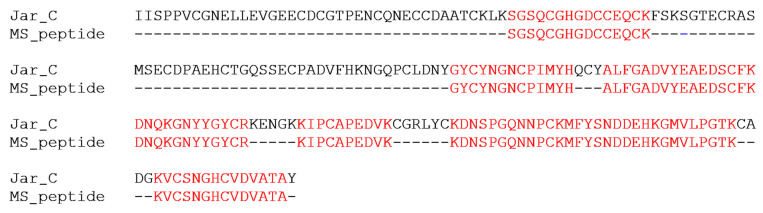
Alignment of the recombinant JarC peptides with the native JarC sequence. The predicted amino acid sequence of recombinant JarC was compared with the native JarC sequence (AAB30855.1) from the venom of *Bothrops jararaca*. The sequences were aligned with ClustalW (web.expasy.org), with identical residues to the first sequence highlighted in red.

The proteomic analysis confirmed that the recombinant protein is JarC, as shown in Figure 2. The identified peptides mapped across the entire sequence show 52% coverage, further confirming the successful expression and accurate identification of the recombinant protein.

### 2.2. rJarC Conformation Is Important for Antibody Recognition

**Figure 3:**
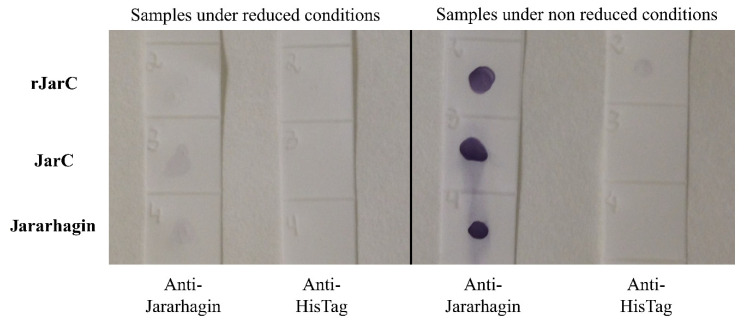
Dot blotting of native (JarC and Jararhagin) and recombinant proteins (rJarC) recognized by a polyclonal anti-jararhagin or anti-histidine antibody under reduced (10 µM Dithiothreitol—DTT) and non-reduced conditions.

The polyclonal anti-jararhagin antibody detected the rJarC, native JarC, and jararhagin only under non-reduced conditions, revealing that the conformational structure is important and was preserved on the recombinant protein (Figure 3).

### 2.3. Native and Recombinant JarC Exhibit Non-Toxic Effects on HUVEC Cells

**Figure 4:**
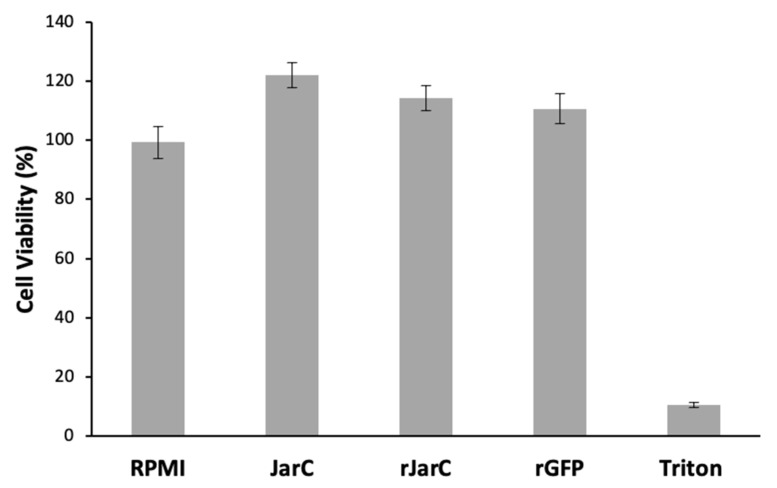
Viability of HUVECs treated with native JarC and rJarC. Cell viability was determined using the MTT assay. HUVECs were not treated (RPMI) or treated with native JarC; rJarC; or rGFP at concentrations of 1 µM or Triton X 100 1% for 24 h.

The results shown in Figure 4 demonstrate that both native and recombinant JarC are non-toxic to HUVEC cells, as shown by the MTT cell viability assay. Here, the recombinant green fluorescent protein (GFP) was used as an unrelated recombinant protein control.

### 2.4. Native and Recombinant JarC Promote HUVEC Cell Migration on Collagen Substrates

**Figure 5:**
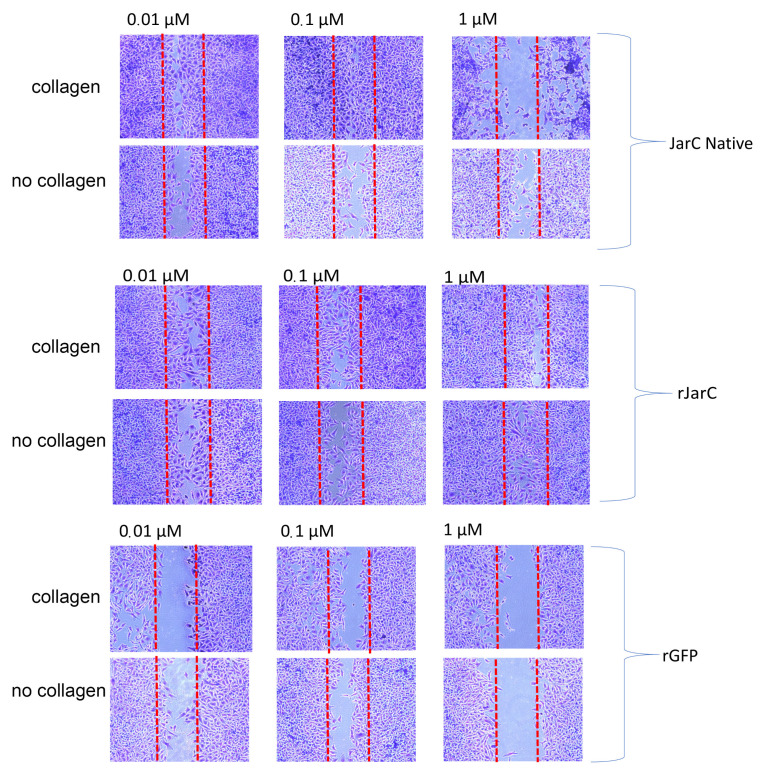
Cell migration induced by JarC and rJarC in vitro (wound healing assay): HUVEC cells CRL1730 were cultured in a 24-well plate coated with collagen or without collagen (no collagen) until they reached approximately 100% confluence, and then a line was drawn down the center of the wells with a 200 µL tip. The cells in culture were treated with native JarC, rJarC, or rGFP at concentrations of 0.01 µM, 0.1 µM, and 1 µM. After 24 h, the culture medium plus the proteins were removed and the cells were fixed and stained with the Protocol Hema 3 staining kit. The plates were examined under a microscope and images were captured using a CCD camera (Leica Microsystems, DFC 310 FX, Mannheim, Germany) coupled to an inverted microscope (Leica Microsystems, DMIL LED, Mannheim, Germany).

Both native JarC and rJarC were effective in stimulating the migration of HUVEC cells attached to a type I collagen substrate or directly on the culture plate surface (Figure 5). Lower doses of native JarC (0.1 µM) were more efficient in promoting cell migration on the collagen-coated surface compared to the untreated surface, while higher doses (1 µM) appeared to induce cell detachment. In contrast, rJarC stimulated cell migration at all tested doses on both substrates, with no significant differences observed in the migration patterns. The rGFP protein, on the other hand, did not stimulate HUVEC cell migration. These results collectively demonstrate the ability of rJarC to induce in vitro cell migration, which is highly similar to the effects observed with the native JarC protein, suggesting that the recombinant protein retains the pro-migratory properties of the native toxin.

## 3. Discussion

Recombinant disintegrins have been extensively studied to elucidate their role in biological processes mediated by integrin receptors, such as hemostasis, proliferation, angiogenesis, and tumor progression [16,29].

Moura-da-Silva et al. [30] previously investigated the expression and functional properties of the recombinant disintegrin- and cysteine-rich domain of jararhagin (JD9) from the venom of *Bothrops jararaca*. In their study, the vector system pET32 was used and a protein yield of 850 µg/L was achieved. In contrast, our study used the bicistronic vector system pSUMOUlp1, which resulted in a significantly higher yield of 5 mg/L culture, illustrating the benefits of optimizing the expression system for better solubility and yield. Both studies confirmed that the structural integrity and immunogenicity of the recombinant proteins were maintained, as evidenced by effective recognition by antibodies. While JD9 inhibited platelet aggregation with an IC50 of 8.5 µg/mL, neither recombinant nor native JarC inhibited collagen-induced platelet aggregation at concentrations of up to 34 µg/mL in our experiments (Supplementary Material). This discrepancy could be due to differences in experimental protocols or purification methods. Nevertheless, recombinant JarC showed remarkable biological activity by promoting cellular mechanisms important for wound healing, indicating several functional properties.

A disintegrin-like toxin from *Bothrops leucurus* with 99% sequence identity to JarC was expressed in *Pichia pastoris* [31], although the yield was not reported because the SDS-PAGE gel was stained with silver, suggesting a low yield. Therefore, the successful expression and purification of recombinant Jar-C (rJarC) using the bicistronic pSUMOUlp1 vector system is an important advance. Simultaneous expression of the Ulp1 protease in this vector enabled the removal of SUMO from JarC, and after a single purification step, a good yield of this disintegrin-like toxin was obtained for further biological characterization.

Since JarC has no predicted glycosylation sites, it is well-suited for expression in bacterial systems. However, its composition with 28 cysteines (13.2% of its amino acid content) poses a challenge for proper folding in the reducing environment of the bacterial cytoplasm. This often leads to misfolding and aggregation, resulting in low yields [32]. Previous attempts to clone and express bothropasin, a metalloproteinase from the venom gland of *Bothrops jararaca* with 39 cysteines, using bacterial, yeast, and mammalian systems, failed to produce sufficient quantities for zymogen activation analysis [33]. Successful expression was achieved only when the disintegrin-like and cysteine-rich domain, which is 99% identical to JarC, was used, reaching 1 mg/L in bacterial cultures. In contrast, the pSUMOUlp1 vector proved to be very effective in expressing the disintegrin-like and cysteine-rich domain of jararhagin (rJarC) in soluble form, achieving a yield of 5 mg/L in bacterial cultures. The correct folding of rJarC is confirmed by its recognition by polyclonal anti-jararhagin antibodies under non-reducing conditions, indicating that the native tertiary structure is preserved, a crucial factor for the functional properties of the toxin.

In our study, both the native and recombinant forms of JarC showed no toxic effect on HUVEC endothelial cells, which is consistent with previous reports on the effect of disintegrin-like/cyst-rich proteins on endothelial cells, such as Alternagin-C, which is derived from the venom of *Bothrops alternatus* [34]. The ability of rJarC to stimulate cell migration on collagen substrates (e.g., in wound healing in vitro assay), which is similar to the migration-promoting properties of the native toxin, suggests that the recombinant protein retains the key functional activities associated with JarC. This is consistent with studies demonstrating the role of Alternagin-C in promoting cell migration [35].

Our results (Supplementary Material) demonstrate that the conformation of the native molecule of jararhagin (SVMP-PIII class) is essential for inhibiting collagen-induced platelet aggregation. Although some studies (e.g., Usami et al., 1994 [6]) report that native JarC can inhibit this aggregation, we did not observe this in our experiments, where both native and recombinant JarC showed no ability to inhibit collagen-induced platelet aggregation. Usami et al. [6] noted that JarC’s inhibitory activity is highly conformation-dependent, as it disappears after reduction and S-carboxyamidomethylation. This suggests that structural integrity is crucial for its function. Possible reasons for these inconsistencies include differences in protein purity. For example, even the smallest traces of jararhagin contamination in the JarC sample could cause an inhibitory effect. However, our findings reveal that both native and recombinant JarC can perform other biological activities, such as promoting cellular events involved in wound healing, even at low doses, marking the first report of their effectiveness in this model.

In contrast, Sánchez et al. [36] demonstrated that r-mojastin 1, a recombinant disintegrin from *C. s. scutulatus* venom, effectively inhibited platelet adhesion and aggregation, highlighting the potential of recombinant disintegrins for therapeutic applications. The differences in activity between rJarC and r-mojastin 1 could be attributed to structural variations, such as the presence of the RGD motif in r-mojastin 1, which is known for its high affinity for integrins. This comparison underscores the importance of structural integrity and optimized expression systems in maintaining the biological activity of recombinant disintegrins. Additionally, Suntravat et al. [37] reported that recombinant colombistatins, disintegrin-like domains from *Bothrops colombiensis*, effectively inhibited platelet aggregation and melanoma cell adhesion, suggesting their potential in therapeutic applications for cancer and thrombotic diseases. This illustrates the variability in the activity of different recombinant disintegrins and emphasizes the importance of structural considerations and expression systems for achieving the desired biological functions.

The study by Minea et al. [38] highlighted the challenges associated with the production of disulfide-rich proteins such as vicrostatin (VCN) in bacterial systems. Using Origami B (DE3) *E. coli*, a strain specifically engineered to support the correct folding of disulfide-rich proteins, VCN, a chimeric recombinant disintegrin, was successfully expressed at significant levels. This underscores the need for a well-optimized expression system to maintain structural integrity, which is essential for maintaining biological functionality.

The work of Minea et al. [38] shows that despite their inherent difficulties, recombinant disintegrins can be produced effectively and retain their functional properties, such as integrin-binding specificity. This is of particular importance as it illustrates the potential of recombinant disintegrins such as rJarC for therapeutic applications such as inhibition of tumor growth and angiogenesis, even though their inhibition of platelet aggregation differs from the native form.

Key limitations of this study include the lack of in vivo validation to assess the translation of the in vitro results. Therefore, addressing this limitation in future research would improve the understanding of the recombinant toxin and its similarities to the native form, which is critical for evaluating its potential therapeutic applications.

## 4. Conclusions

In summary, the use of the bicistronic pSUMOUlp1 vector system facilitated the efficient expression and purification of rJarC and provided sufficient amounts of this disintegrin-like toxin for comprehensive studies on its biological activities. The recombinant protein retained essential functional properties, including its ability to promote one-dimensional cell migration, comparable to native JarC. These results form the basis for further research into the therapeutic potential of this recombinant toxin, particularly in the areas of wound healing, angiogenesis, and extracellular matrix remodeling.

## 5. Material and Methods

### 5.1. Cloning of JarC into the pSUMOUlp1 Vector

The JarC sequence (1077 to 1713 bp) from the GenBank database (X68251.1) was codon-optimized and synthesized by GeneArt (Thermo Fisher Scientific, Waltman, MA, USA) for efficient expression in E. coli. The gene was first inserted into the commercial vector pUC57. To subclone the JarC sequence into the pSUMOUlp1 vector, it was amplified from pUC57 by polymerase chain reaction (PCR) using the primers JarC_Forward: 5′ggatccATTATTTCACCTCC3′ and JarC_Reverse: 5′ctcgagAAGCTTGTAGCC3′, which contain BamHI and XhoI restriction sites (underlined), respectively. After amplification, the JarC-PCR product and the pSUMOUlp1 vector were digested with the restriction enzymes BamHI and XhoI (Thermo Fisher Scientific, Waltman, MA, USA). The digested fragments were electrophoresed on a 1% agarose gel (Sigma-Aldrich Co., St. Louis, MO, USA), and the bands corresponding to JarC and pSUMOUlp1 were excised and purified using the Wizard^®^ plus Miniprep DNA purification system kit (Promega^®^, Madison, WI, USA) according to the manufacturer’s guidelines. For ligation, 25 μg of vector and 75 μg of insert were combined in a 1:3 molar ratio along with 1 μL ligase, 2 μL ligase buffer (50 mM Tris-HCl, pH 7.5; 10 mM MgCl_2_; 10 mM dithiothreitol; 1 mM ATP; and 25 µg/mL BSA) to obtain a total reaction volume of 15 µL, which was incubated overnight at 4 °C.

### 5.2. rJarC Expression in pSUMOUlp1 Vector

The pSUMOUlp1 vector was introduced into competent Escherichia coli BL21 Star (DE3) cells by thermal shock transformation. After transformation, 1 mL of Luria-Bertani medium (LB medium) was added to the bacteria and incubated in a dry bath at 37 °C with shaking at 400 rpm for 1 h. Subsequently, 1 mL of the bacterial culture was transferred to 30 mL of LB medium containing chloramphenicol (34 mg/mL) and 1 mM calcium chloride and magnesium chloride. This culture was incubated overnight at 30 °C with shaking at 180 rpm. The next day, 4 mL of the preculture was inoculated into 200 mL of LB medium and grown at 30 °C with shaking at 180 rpm until bacterial growth reached an optical density of 0.5–0.6 at 600 nm. At this point, the expression of rJarC was induced with 1 mM isopropyl-β-D-thiogalactopyranoside (IPTG) (Sigma-Aldrich Co., St. Louis, MO, USA.) for 4 h. After induction, the bacterial culture was centrifuged at 12,000× *g*, and the pellet was stored at −20 °C for further processing. The pellet was later resuspended in wash and equilibration buffer (20 mM sodium phosphate, 500 mM NaCl, and 20 mM imidazole, pH 7.40), followed by the addition of lysozyme (Sigma-Aldrich Co., St. Louis, MO, USA) at a final concentration of 0.2 mg/mL. The mixture was homogenized for 30 min at room temperature. The bacterial suspension was then sonicated in an ice bath using an intermittent program (20% amplitude with 3 s pulses and 4 s intervals), with a 4 min cooling break between cycles. This sonication procedure was repeated five times. The lysate was then centrifuged at 12,000× *g* and 4 °C for 15 min. The resulting supernatant was used to purify the recombinant protein.

### 5.3. Purification of rJarC

Purification was performed by Immobilized Metal Affinity Chromatography (IMAC) with Ni Sepharose^®^ 6 Fast Flow Resin (GE^®^ Healthcare, Boston, MA, USA) according to the manufacturer’s protocol. The supernatant was applied to 1.5 mL of pre-washed and equilibrated resin in binding buffer (20 mM sodium phosphate, 500 mM NaCl, and 20 mM imidazole, pH 7.40). Protein binding to the resin was achieved by incubation in a carousel homogenizer for 30 min. After binding, the resin was washed twice with 70 mM imidazole, and the bound proteins were eluted with 1M imidazole. The eluate was dialyzed overnight at 4 °C in phosphate-buffered saline (PBS) containing 1 mM NaCl and 1 mM MgCl, pH 7.40. The protein was then separated on a 12% polyacrylamide gel under reducing conditions using sodium dodecyl sulfate (SDS) (Thermo Fisher Scientific, Waltman, MA, USA). Molecular weight estimation was performed using the Page Ruler Unstained Protein Ladder (Thermo Fisher Scientific, Waltman, MA, USA). The gel was stained with Coomassie blue (40% methanol, 7% glacial acetic acid, and R-250 dye), and the protein concentration was measured using the Bicinchoninic Acid (BCA) method (Thermo Fisher Scientific, Waltman, MA, USA).

### 5.4. In-Solution Digestion and Proteomic Analysis

The volume of 50 μL containing 25 µg rJarC was subjected to in-solution digestion in the following steps: (1) 5 μL of 100 mM dithiothreitol (DTT; Sigma-Aldrich, Co., St. Louis, MO, USA) in 50 mM ammonium bicarbonate was added and incubated for 30 min at 60 °C; (2) 2.5 μL of 200 mM iodoacetamide (IAA; Sigma-Aldrich, Co., St. Louis, MO, USA) in 50 mM ammonium bicarbonate was then added and the sample was incubated for 30 min at room temperature in the dark; and (3) digestion was performed with 10 μL of trypsin (40 ng/μL in 100 mM ammonium bicarbonate) for 12 h at 22 °C. The reaction was terminated with 50% ACN/5% TFA. The sample was analyzed using a liquid chromatography-mass spectrometry system comprising an ESI-IT-TOF mass spectrometer coupled to a UFLC (20A Prominence, Shimadzu, Kyoto, Japan). Each sample was injected into a Kinetex C18 column (5 μm, 50 × 2.1 mm) and separated using a binary solvent system: (A) water:DMSO:acid (949:50:1) and (B) ACN:DMSO:water:acid (850:50:99:1). A gradient of 0–40% solvent B was applied for 35 min at a flow rate of 0.2 mL/min. A Shimadzu SPD-M20A PDA detector monitored the samples prior to mass spectrometry injection.

The interfacial voltage was set to 4.5 kV, with a capillary voltage of 1.95 kV at 200 °C. Fragmentation was induced by argon with a collision energy of 55%. MS spectra were recorded in positive ion mode over a mass range of 350–1400 *m*/*z*, while MS/MS spectra were recorded in a range of 50–1950 *m/z*. The raw data from the Shimadzu LCD (LCMS Protein Postrun, Shimadzu) were converted to .mzXML format for analysis. The converted file (named E.Seg1Ev1.mzXML) is included in the Supplementary Material. Data processing, including de novo peptide sequencing and proteomic identification, was performed using Peaks Studio V7.0 software (BSI, Toronto, ON, Canada). Parameters for proteomic identification included a mass error tolerance of 0.1 Da for both MS and MS/MS, methionine oxidation as a variable modification, carbamidomethylation as a fixed modification, trypsin as an enzyme, up to three missed cleavages, a maximum of three variable post-translational modifications (PTMs) per peptide, and one non-specific cleavage. The samples were compared with the database “Animal Toxins”, which was compiled from UniProt (https://www.uniprot.org/ accessed on 22 August 2024) and contains 164,244 entries.

### 5.5. Western Blot Analysis

For the Western blot analysis with anti-His antibodies, samples of recombinant JarC were analyzed on a 12% SDS-PAGE under reducing conditions. Subsequently, the samples were transferred to nitrocellulose membranes using the Trans-Blot^®^ SD Semi-Dry Transfer Cell (Bio-Rad^®^ Laboratories, Hercules, CA, USA) following the manufacturer’s recommendations. After transfer, the nitrocellulose membranes were stained with Ponceau S^®^ (Merck Millipore Corporation, Darmstadt, Germany) 1:20 to verify the transfer of the proteins. To remove the dye, the membranes were washed with TBS-Tween (20 mM Tris, 150 mM NaCl, 0.05% Tween 20, pH 7.5) until complete removal. Subsequently, the membranes were blocked with incubation buffer (Tris/NaCl, pH 7.5 with 5% powder skimmed milk) for 2 h at room temperature and then washed 3 times with TBS-Tween. Afterward, the membranes were incubated for 2 h with mouse monoclonal anti-polyhistidine antibody (Sigma Life Science, Merck Corporation, Darmstadt, Germany) at a 1:1000 dilution in an incubation buffer. Afterward, the membranes were washed with TBS-Tween and incubated for 2 h with the peroxidase-labeled anti-mouse IgG (Sigma Life Science, Merck Corporation, Darmstadt, Germany) at a 1:5000 dilution in incubation buffer. Then, a new wash cycle was performed and the antigenic components were revealed with 0.05% (*w/v*) 4-chloro-1α-naphthol in 15% (*v/v*) methanol in the presence of 0.03% H_2_O_2_ (*v/v*).

### 5.6. rJarC Detection by Polyclonal Anti-Jararhagin Antibodies (Dot Blot)

The structural similarity between rJarC and native JarC was investigated using an anti-jararhagin antibody in a dot-blot assay. This assay was performed under both reducing and non-reducing conditions to evaluate conformational features. For reducing conditions, 2 µg of each sample was mixed with 5 µL of sample buffer containing a reducing agent (2.5% DTT, 62.5 mM Tris at pH 6.8, 10% glycerol, 2% SDS) and heated at 100 °C for five minutes. For analysis under non-reducing conditions, 2 µg of each sample was added to 5 µL of PBS and applied directly to the nitrocellulose membrane. The following proteins were used: rJarC, JarC, and Jararhagin. These proteins were diluted in PBS and the concentration was adjusted to 2 µg/5 µL.

A total volume of 5 µL of each sample was applied directly to the nitrocellulose membrane and dried at 37 °C. The membranes were then blocked in Tris-phosphate buffered saline (TBS) containing 5% skim milk powder (Molico^®^-Nestle) for 2 h with gentle agitation, followed by three 10 min washes with 0.1% TBS/Tween 20. After blocking, the membranes were incubated overnight at 4 °C with one of two primary antibodies: a rabbit polyclonal anti-jararhagin antibody diluted 1:1000, or a commercially available anti-histidine antibody (Sigma-Aldrich Co., St. Louis, MO, USA) prepared in mice and diluted 1:3000 in TBS containing 5% skim milk. The next day, the membranes were washed three times for 10 min with 0.1% TBS/Tween 20 and then incubated with secondary antibodies—either peroxidase-labeled anti-rabbit IgG or anti-mouse IgG diluted 1:1000 in TBS containing 5% skim milk—for 2 h with agitation. After a final series of three 10 min washes with TBS-Tween, peroxidase activity was visualized with a chromogenic substrate solution containing 4-chloro-1-naphthol (Sigma-Aldrich Co., St. Louis, MO, USA) and H_2_O_2_ prepared according to the manufacturer’s instructions. The reaction was stopped with distilled water as soon as the bands became visible on the nitrocellulose membrane.

### 5.7. Purification of Native JarC (JarC)

Native JarC was isolated from freeze-dried *Bothrops jararaca* venom provided by the Herpetology Laboratory of the Butantan Institute according to the previously established protocol [14]. The protein concentrations of native and recombinant JarC (rJarC) were determined using the Bradford assay (Bio-Rad^®^ Laboratories, Hercules, CA, USA) according to the manufacturer’s guidelines. The purity of the proteins was determined by SDS-PAGE under denaturing conditions according to the previously described method [39]. For analysis, toxins were resolved on a 15% SDS-PAGE gel at 110 V and 55 mA for approximately 3 h and 10 min using the PageRuler Unstained Protein Ladder (Thermo Fisher Scientific, Waltman, MA, USA) as a molecular mass marker. The gel bands were stained with a solution containing 0.2% (*w*/*v*) Coomassie Blue R-250 (Thermo Fisher Scientific, Waltman, MA, USA) prepared in a 1:1 (*v*/*v*) mixture of water and methanol, and then decolorized with a solution of methanol and distilled water containing 30%/10% acetic acid.

### 5.8. HUVEC Cell Culture

Human vascular endothelial cells (HUVEC) of the ATCC—CRL1730 line were grown in RPMI 1640 culture medium (Gibco^®^) supplemented with 10% complement-inactivated fetal bovine serum (FBS), 2 mM L-glutamine (Gibco^®^), 100 U/mL penicillin/100 µg/mL streptomycin (Gibco^®^) in an incubator at 37 °C and 5% CO_2_. The culture medium supplemented with 10% FBS was used only for the maintenance and proliferation of the cells and was replaced during the experiments by a culture medium supplemented only with antibiotics and L-glutamine. After reaching 80–100% confluence, the cells were harvested with trypsin/EDTA (Gibco^®^) according to the manufacturer’s instructions.

### 5.9. Cell Viability Assay

A total of 20,000 CRL1730 HUVEC cells per well, diluted in RPMI supplemented with 10% FBS, were seeded in a 96-well plate. These cells were kept at 37 °C for 2 h in the presence of CO_2_ to adhere to the bottom of the plate. Then, the culture medium was removed, the cells were washed twice with PBS buffer, and RPMI culture medium was added. Cells were incubated for 24 h in the presence of 1 µM JarC, rJarC, rGFP (recombinant green fluorescent protein used as an unrelated recombinant protein), or 0.1% Triton X-100 (a negative control toxic to the cells). RPMI medium supplemented with 10% FBS was used as a positive control. For the MTT (3-(4,5-dimethylthiazolyl-2)-2,5-diphenyltetrazolium bromide) assay, MilliporeSigma™ Chemicon™ MTT Cell Growth Assay Kit (Temecula, CA, USA) was used according to the manufacturer’s instructions. Finally, the plate was analyzed with a SpectraMax M-Series Microplate Reader (Molecular Devices, San José, CA, USA) at 570 nm.

### 5.10. In Vitro Migration of Endothelial Cells: Wound Healing Assay

The assay to test cell movement in an empty field mimicking an in vitro model of wound healing was performed using CRL 1730 HUVEC cells as described in [40,41]. An amount of 1 × 10^6^ CRL1730 HUVEC cells per well, diluted in RPMI supplemented with 10% FBS, was seeded into a 24-well culture plate that had been previously sensitized with collagen type I (50 µg/mL) or had not received any sensitization treatment. The cells were maintained in complete RPMI medium at 37 °C and 5% CO_2_ for 24 h to attach to the substrate. After this time, the cells were washed twice with PBS buffer, RPMI culture medium containing only the antibiotics was added, and a line was scraped into each well using a sterile tip (200 µL). Samples of JarC and rJarC were added to these wells at concentrations of 0.01 µM, 0.1 µM, and 1 µM, or RPMI medium. The recombinant EGFP protein was used as an unrelated recombinant protein control. After 24 h, cells were fixed and stained using the HEMA 3 Stain Set Protocol (Thermo Fisher Scientific, Waltman, MA, USA) according to the manufacturer’s recommendations and analyzed under an inverted microscope with phase contrast (LEICA). Images were captured in triplicate using a CCD camera attached to the microscope in 6 central fields for each sample. A representative field was selected to create the image. The presence of cells within the scratched area indicates whether the cell migration process has taken place.

## Data Availability

The original contributions presented in the study are included in the article and Supplementary Material, further inquiries can be directed to the corresponding author.

## Supplementary Materials

The following supporting information can be downloaded at: https://www.mdpi.com/article/10.3390/toxins16120524/s1, Figure S1: Collagen-induced platelet aggregation assay.
