# Supplementary material for: Bicistronic Vector Expression of Recombinant Jararhagin-C and Its Effects on Endothelial Cells
Source: Toxins (Basel). 2024 Dec 3;16(12):524. doi: 10.3390/toxins16120524 (PMC11728617; doi:10.3390/toxins16120524)
Supplement: Supplementary file 1 [file toxins-16-00524-s001.zip › toxins-3267544-supplementary.pdf]

toxins

MDPI

Supplementary Materials

Bicistronic Vector Expression of Recombinant Jararhagin-C and Its Effects on Endothelial Cells

Karla Fernanda Ferraz [1], Lhiri Hanna De Lucca Caetano [1], Daniele Pereira Orefice [1], Paula Andreia Lucas Calabria [1], Maisa Splendore Della-Casa [1], Luciana Aparecida Freitas-de-Sousa [1], Emidio Beraldo-Neto [2], Sabri Saeed Sanabani [3], Geraldo Santana Magalhães [1], Patricia Bianca Clissa [1,*]

[1] Immunopathology Laboratory, Butantan Institute, São Paulo 05585-090, Brazil; karlafernandaferraz@gmail.com (K.F.F.); lhiri.hanna@gmail.com (L.H.D.L.C.); daniele.orefice@butantan.gov.br (D.P.O.); paula.calabria@fundacaobutantan.org.br (P.A.L.C.); maisa.casa@butantan.gov.br (M.S.D.-C.); luciana.sousa@butantan.gov.br (L.A.F.-d.-S.); geraldo.magalhaes@butantan.gov.br (G.S.M.)

[2] Biochemistry Laboratory, Butantan Institute, São Paulo 05503-900, Brazil; emidio.beraldo@butantan.gov.br

[3] Laboratory of Medical Investigation LIM-56, Division of Dermatology, Medical School, University of São Paulo, Sao Paulo 05508-220, Brazil; sabri.sanabani@hc.fm.usp.br

* Correspondence: patricia.clissa@butantan.gov.br

Material and Methods

Collagen-induced platelet aggregation

Fresh human citrated blood from healthy donors was collected after approval by the Ethics Committee for Research of the São Paulo Department of Health (3.101.898), CAAE: 02990818.3.0000.0086, and centrifuged at 800 xg for 20 minutes at room temperature to obtain platelet-rich plasma (PRP). The PRP was then centrifuged at 2000 xg for 15 minutes and the platelets were resuspended in wash buffer, followed by a further centrifugation at 2000 xg for 15 minutes. The supernatant was discarded and the procedure was repeated. The platelets were resuspended in Tyrode's buffer (138 mM NaCl, 3 mM KCl, 1 mM MgCl2, 1 mM glucose, 0.5 mM NaH2PO4, 20 mM Hepes, pH 7.4).

To study the effects of native (jararhagin and jararhaginC) and recombinant jararhaginC, three different proteins doses were used. For this, 90 µL of proteins solution (2.0, 4.0 and 8.0 µM) was incubated with 400 µL of platelets for 3 minutes at 37°C. Subsequently, 10 µL of 2 mg/mL collagen (Chrono-Log Corporation) was added. The final concentration of toxins used were up to 34 µg/ml or 1.5 µM. Platelet aggregation was measured using an Aggregometer Cronolog (model 490-2D) and expressed as the percentage increase in light transmission, with 100% being the value for platelets incubated with control buffer and stimulated with collagen under the same conditions.

Results

rJarC and native JarC do not inhibit collagen-induced platelet aggregation.

The platelet aggregation inhibition assay was conducted using washed platelets incubated with 2 mg/mL collagen (**Supplementary figure S1A**). All toxins were tested at the same molar final concentration. The results express the dose of 1.5 µM. The native Jararhagin molecule, which includes the metalloproteinase/cysteine-rich and disintegrin-like domains, completely inhibited collagen-induced platelet aggregation (**Supplementary figure S1B**). In contrast, both native JarC (**Supplementary figure S1C**) and recombinant JarC (**Supplementary figure S1D**) did not inhibit collagen-induced platelet aggregation.

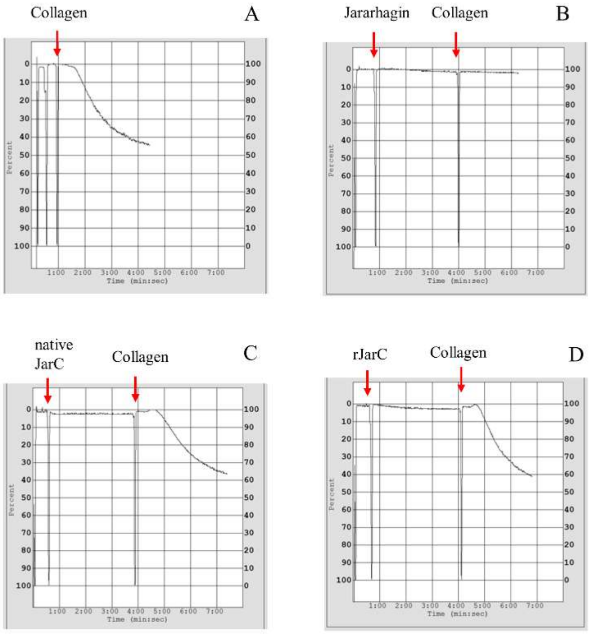

Supplementary figure S1: Collagen-induced platelet aggregation assays. (A) Platelets incubated with collagen alone. (B) Complete inhibition by native Jararhagin (1.5 μM**)**. (C) 1.5 μM of native JarC and (D) recombinant JarC (rJarC) show no inhibition of platelet aggregation. Arrows indicate the addition of proteins and collagen.
